# Genuine-field modeling of partially coherent X-ray imaging systems

**DOI:** 10.1107/S1600577520006979

**Published:** 2020-07-10

**Authors:** Antonie Verhoeven, Christian Hellmann, Frank Wyrowski, Mourad Idir, Jari Turunen

**Affiliations:** a Institute of Photonics, University of Eastern Finland, PO Box 111, 80101 Joensuu, Finland; b Wyrowski Photonics UG, Kahlaische Straße 4, D-07745 Jena, Germany; c Institute of Applied Physics, Friedrich-Schiller University, Albert-Einstein-Straße 15, D-07745 Jena, Germany; d Brookhaven National Laboratory, New York, USA

**Keywords:** partial spatial coherence, wavefront propagation, beamline design, non-ideal mirrors

## Abstract

A genuine representation of the cross-spectral density function as a superposition of mutually uncorrelated, spatially localized modes is applied to model the propagation of spatially partially coherent light beams in X-ray optical systems. Numerical illustrations based on mode propagation with *VirtualLab* software are presented for imaging systems with ideal and non-ideal grazing-incidence mirrors.

## Abstract 2

A genuine representation of the cross-spectral density function is applied to model partially coherent wave propagation in X-ray imaging systems.

## 1. Introduction   

Since synchrotron radiation (SR) is a random process, statistical optics is required for its proper description. Models for spatial coherence of SR based on second-order coherence theory have indeed been developed (Geloni *et al.*, 2008 ▸; Vartanyants & Singer, 2016 ▸; Glass, 2017 ▸), which allow the calculation of the cross-spectral density (CSD) function for a variety of present and future X-ray sources with different degrees of coherence (Vartanyants & Singer, 2010 ▸). With such source models available, it is of interest to model X-ray optical systems (beamlines) including imaging systems with imperfect (aberrated) focusing mirrors.

In general, CSD is a four-dimensional (4D) function and its direct propagation in optical systems therefore requires 4D integrals (Fourier transforms). The use of such direct techniques requires, in practice, that the entire problem can be treated as separable in two orthogonal transverse spatial directions. In this case the dimensionality reduces from four to two, but the assumption excludes full analysis of system aberrations. A similar reduction of dimensionality is achieved, without requiring separability, if the CSD is represented as an incoherent superposition of mutually orthogonal coherent modes, which are the eigenmodes of a Fredholm differential equation with the CSD as a kernel (Wolf, 1982 ▸). Such a Mercer-type coherent-mode expansion is fully general, and it has proven particularly convenient for the description of X-ray free-electron laser (FEL) radiation: the high degree of coherence then implies that only a few coherent modes are needed to describe the CSD accurately (Roling *et al.*, 2011 ▸; Singer *et al.*, 2012 ▸; Vartanyants *et al.*, 2011 ▸). In the case of low-coherence X-ray fields, however, a larger number of modes are required. While the Mercer modes can be determined numerically (Glass & Sanchez del Rio, 2017 ▸), the high-order modes tend to be highly oscillatory and are therefore not very attractive in numerical computations.

In this paper we introduce and demonstrate a model for X-ray imaging systems based on the genuine representation of the CSD. Like the (Mercer) coherent-mode decomposition, this representation is fully general: the CSD is expressed as an incoherent superposition of (generally non-orthogonal) modal fields (Gori & Santarsiero, 2007 ▸; Gori *et al.*, 2009 ▸; Martínez-Herrero & Mejías, 2009 ▸; Martínez-Herrero *et al.*, 2009 ▸; Shirai, 2009 ▸; Khubbutdinov *et al.*, 2019 ▸). Considering the interpretation of Gori & Santarsiero, such modal fields can be viewed as impulse responses of an arbitrary imaging system with incoherent illumination. Hence, in particular, the field in the image plane of a condenser system with an incoherent primary X-ray source (Chang *et al.*, 2003 ▸) has precisely the form of a genuine representation. However, since the representation is fully general, it applies to sources and fields of any state of coherence, including third-generation and future high-emittance sources (Geloni *et al.*, 2008 ▸).

In the special case of fields with a Schell-model angular correlation function (Schell, 1967 ▸), the modal fields are identical, spatially shifted ‘elementary’ modes (Gori & Palma, 1978 ▸; Gori, 1980 ▸; Vahimaa & Turunen, 2006 ▸; Tervo *et al.*, 2010 ▸; Turunen, 2011 ▸) that can be spatially well confined. In the model of Coïsson (Coïsson, 1995 ▸), where the source-plane electron position and angular distribution are approximated as Gaussian functions, the elementary modes are also Gaussian functions with weights obeying the Gori–Palma model (Gori & Palma, 1978 ▸). Such Gaussian-beam superpositions have indeed been recently exploited, to some extent, to calculate image-plane intensity distributions in low-coherence X-ray imaging systems (Canestrari *et al.*, 2014 ▸; Shi *et al.*, 2014 ▸; Rakitin *et al.*, 2018 ▸). Here, in addition to intensity distributions, we also consider the spatial coherence of the image-plane fields.

The genuine approach, based on identical elementary modes, has been used to describe beam shaping (Singh *et al.*, 2013 ▸) and imaging (Singh *et al.*, 2015 ▸) systems in the visible spectral region. In this region conventional optical systems are essentially space-invariant over a considerable spatial domain, typically far in excess of the spatial extent of the elementary modes. We will see that the situation is quite different in X-ray systems with Kirkpatrick–Baez (KB) mirrors, where the space-invariant region of the system is of the same order of magnitude as the spatial extent of the modes.

We begin with a description of the genuine field representation (Section 2), which we apply to model an X-ray imaging setup consisting of two elliptical grazing-incidence KB mirrors with surface-shape errors. We define the SR source using the anisotropic Gaussian Schell model (DeSantis *et al.*, 1980 ▸; Li & Wolf, 1986 ▸), though our approach readily permits the use of more sophisticated source models. The implementation of the field propagation technique that propagates the modes through the optical system is described in Section 5. Numerical results for image-plane modes as well as the total partially coherent images and image-plane correlation functions are given in Section 6.

## 2. The genuine field representation   

Throughout this paper we will describe the spatial coherence of SR with the aid of a scalar CSD evaluated at the central frequency of the spectrum. This approach is standard in the literature (Geloni *et al.*, 2008 ▸) but its validity is not *a priori* obvious since SR sources are non-stationary (pulsed) and electromagnetic. The conditions for this description to be applicable are formally discussed in Appendix *A* [App appa]. Within such conditions, and keeping in mind the reservations involved, we denote by 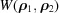 the spatial part of the scalar CSD at points 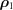 = 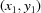 and 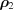 = 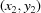 in any transverse plane perpendicular to the nominal propagation direction (*z* axis) of the SR beam.

In general, any physically realizable CSD has a coherent-mode representation (Wolf, 1982 ▸). This decomposition follows from ‘Mercers Theorem’ and requires that *W* is square integrable, 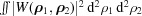 < 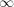, is hermitian, 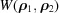 = 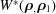, and non-negative definite, 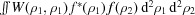 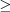 0, for any square integrable function *f*. The first condition follows from the field having a finite extent/finite energy; the second directly follows from the ensemble average over the field realizations; and the third from showing that the cross-spectral density matrix is non-negative definite. The coherent-mode representation reads as
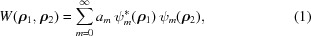
where 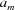 and 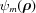 are the eigenvalues and eigenfunctions of the Fredholm integral equation,
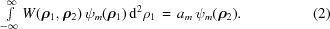
The eigenvalues 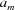 are real and non-negative and the eigenfunctions 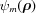, known as the coherent modes of the CSD, are orthogonal in the sense that
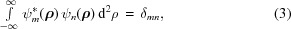
where 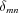 is the Kronecker delta symbol.

Alternatively, the CSD can always be represented in a genuine form (Gori & Santarsiero, 2007 ▸). This requires that the kernel is non-negative definite and as described by the theory on the kernel Hilbert spaces. Given that this is already a required condition for equation (1), the genuine form can therefore also be applied to the CSD here. The genuine representation has the form
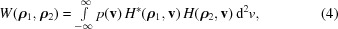
where 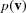 is a non-negative function of a (generally continuous) parameter 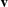 and 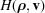 is the kernel of an arbitrary linear transformation. The representations (1) and (4) are both fully general and connected (Martínez-Herrero *et al.*, 2009 ▸). To see this, let us define functions
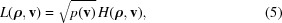
so that equation (4) takes the form
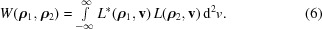
Let us further consider any complete set 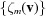 of functions that are orthogonal in the same sense as in equation (3). Writing
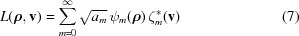
and inserting into equation (6) leads immediately to equation (1), proving the equivalence of these two representations. Unlike the coherent-mode representation, the genuine representation is not unique because of the freedom to choose the set 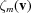.

As a result of the considerations presented above, it is possible (at least in principle) to determine explicit genuine representations of any (known) CSD. This can be accomplished, on one hand, by first solving the Fredholm equation (2) and then determining 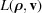 using, for example, equation (7) with the choice 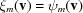. In the case of X-ray fields, the CSD can be modeled starting from primary electron distributions (Glass, 2017 ▸). Fortunately, however, vastly simplified approaches are often adequate (Gori *et al.*, 1998*a* ▸).

The angular correlation function (ACF), which describes correlations between any two spatial frequencies 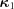 and 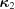, is defined as
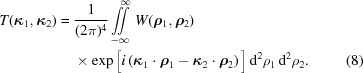
On inserting (6) into (8) we find that the ACF has a genuine representation
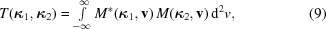
where
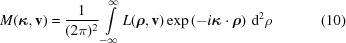
is the two-dimensional spatial Fourier transform of the kernel 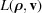.

A particularly attractive form of the genuine representation is obtained if the ACF is of the Schell-model form (Schell, 1967 ▸), *i.e.* if it can be represented as
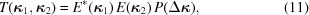
where 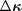 = 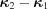, and *E* and *P* are arbitrary, generally complex-valued functions. The angular spectral density (angular intensity) of the field is now given by
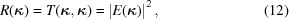
and the angular degree of spatial coherence has the form
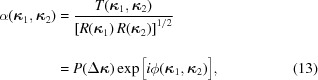
where 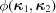 = 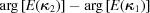.

Inserting (11) into the inverse of (8) and introducing a Fourier representation
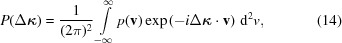
we find that
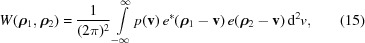
where
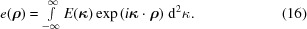
This is of the genuine form of equation (4) with
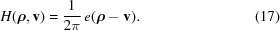
We can now consider the CSD as a superposition of spatially shifted ‘elementary’ fields, all of which have an identical functional form and are weighted by the function 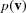. According to equation (16) the elementary field 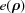 is determined uniquely by the function 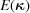 or, up to a phase factor, by the angular intensity 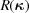 defined in equation (13). In view of the Fourier inverse of equation (14), the weight function 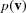 is specified by 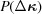.

It is, of course, clear that realistic optical fields are not likely to obey the Schell-model assumption of equation (11) precisely, but in many cases this can be approximately true. Then the functions 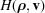 would not all be exactly of the same form as in equation (17), but would nevertheless have nearly the same spatial extent. Moreover, the interpretation of the parameter 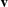 as a ‘center position’ of 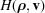 or, equivalently, of the kernel 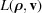, would still be useful. These arguments can be qualitatively substantiated by considering the intuitive interpretation of the genuine field representation presented by Gori & Santarsiero (2007 ▸). They pointed out that fields of the form of equation (4) can be generated (for example, but not exclusively) by imaging an incoherent primary source with an optical system having a point-spread (impulse-response) function 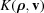, where 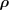 refers to the spatial position at the output plane of the system and 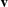 refers to the position of a point source at the input plane. The functional form of the output ‘modal field’ intensity 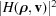 then depends on 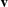. If the system is space-invariant, the only modification of the output-spot is a shift of its center position, which depends uniquely on 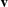. However, even in space-variant systems the output spot would be centered close to this position and be confined nearby. We will see later on, by numerical simulations, that this type of reasoning is valid in X-ray imaging systems.

For sources that are not diffraction limited it is often a good approximation to model SR sources by assuming that the associated CSD is separable in the Cartesian coordinates (Khubbutdinov *et al.*, 2019 ▸),
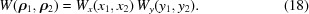
However, the distributions of 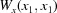 and 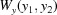 can be very different because of the anisotropic nature of the SR generation process, and the separability is generally broken when the field passes through the SR beamline, as we will see in our numerical simulations. Assuming equation (18) to hold, we can always represent both 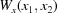 and 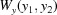 by their coherent-model expansions. Considering the *x*-dependent factor and dropping the subscript *x* for brevity we then have
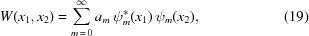
and a similar expansion holds for the *y*-dependent factor.

The most commonly used model field in coherence theory is the so-called Gaussian Schell model (GSM) beam with
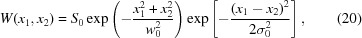
where 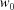 and 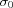 represent the beam width and coherence width, respectively. The angular correlation function is then also of the Schell-model form, *i.e.*

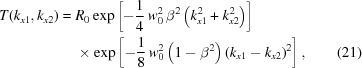
where 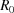 = 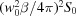 and the parameter
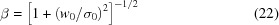
is bounded between zero (incoherent limit 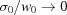) and unity (fully coherent limit 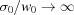). Hence the genuine representation of GSM fields has the form of an elementary-field expansion. Recognizing the forms of 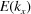 and 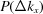 by comparing equations (11) and (21) we find from equation (16) that the (normalized) elementary field is
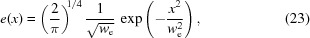
with 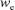 = 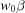. From the inverse of equation (14) we also see that the weight function is
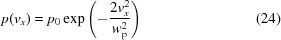
with 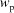 = 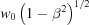 and 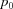 = 
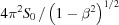
.

For GSM fields the (normalized) coherent modes are the Hermite–Gaussian modes (HG) of the standard spherical-mirror laser resonator, *i.e.*

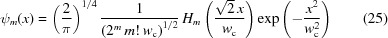
with a transverse scaling parameter 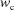 = 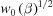 and expansion coefficients that obey the law
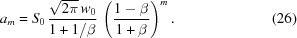
We stress that the Gaussian elementary mode in equation (25) should not be confused with the lowest-order (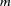 = 0) Gaussian coherent mode in equation (25) since their transverse scales are different for any given GSM field, apart from the fully coherent (single mode) case. For any partially coherent source-plane field, the elementary field is more confined than the fundamental coherent mode. If we assume (as we do in the following) that the the divergence is rotationally symmetric, the source field in the partially coherent case is wider in the partially coherent direction as it is in the coherent (diffraction-limited) direction.

**Figure 1:**
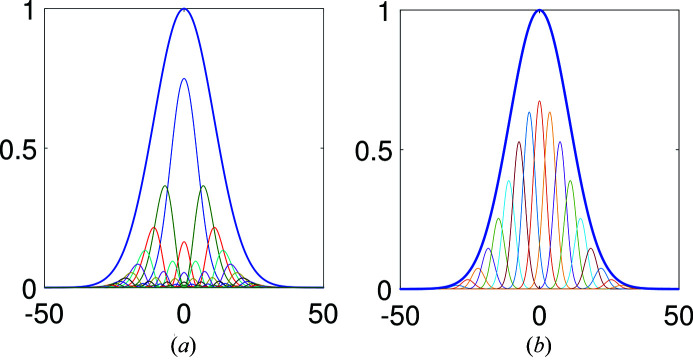
Cross section of the CSD/intensity profile at the source plane represented by either the intensity of the HG modes (*a*) or shifted elementary modes (*b*).

Fig. 1 ▸ shows a cross section of the CSD/intensity profile at the source plane if one either uses the mode expansion equation (1) by means of HG modes equation (25) or a superposition of shifted elementary modes equation (14) with Gaussian elementary modes equation (23).

For the elementary mode representation no explicit equation was found to express the error made when truncating the number of elementary modes. To ensure that the elementary modes properly represent the field, a large enough portion of the weight function 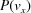 should be sampled by choosing 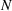 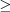 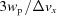. The elementary modes should also at least partially overlap, which is ensured if 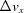 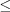 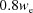.

The advantage of HG mode representation is that fewer modes are typically required to accurately represent the field. For example, in the shown figure the HG representation has a <0.1% error while elementary modes result in a <0.5% error, where error is defined as the total intensity difference between perfect and shown representation in the shown cross section. On the flip side, higher-order HG modes oscillate strongly and thus require more grid-points to accurately represent numerically. The shifted elementary modes are all of the same shape which is also advantageous for an aplanatic optical system, in which the response to each mode is the same so that only one mode needs to be propagated. If the system becomes space variant then a sufficient number of elementary modes are needed to properly sample the system response. The other major difference is that the width of the elementary modes decreases as coherence decreases. This is in particular advantageous when propagating a low-coherence source through an aperture, allowing one to ignore those modes that have no significant energy contribution inside the aperture area.

Suppose we wish to model a beamline similar to the Sub-micron Resolution X-ray (SRX) beamline at NSLS II (De Andrade *et al.*, 2011 ▸) along with a source that has a FWHM beam width of 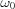 = 22.0 µm × 548 µm and a divergence angle of 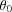 = 12.4 µrad × 24.8 µrad in the horizontal and vertical (H, V) direction, respectively. This source is assumed to obey the simple HG model and has a particular poor coherence along the vertical direction with β ≃ 0.2 × 0.004 (H, V). The supposed setup improves the coherence along the vertical direction by focusing the source along the horizontal direction onto an aperture some 49 m away from the undulator source. For this particular scenario the representation of the source itself would require about 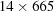 HG modes or about 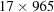 shifted elementary modes. If one supposes that a square aperture of 50 µm × 50 µm is located at the center of the horizontal focus, then only 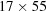 elementary modes need to be considered as all other modes will miss the aperture opening. For this consideration only those modes that have their center within 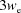 from the closest edge of the aperture at the aperture plane were counted. The lower numerical cost per mode along with a great decrease in number of modes would be a large advantage for such a setup and is a main reason why elementary modes are considered in this paper.

The SRX beamline with inclusion of this aperture along was originally considered in this paper but would require an operator that extracts the quadratic phase from the field after the aperture to facilitate the switch between field propagation operators as described in Section 5. As this operator was yet to be implemented at time of writing this paper, the source model and setup have been simplified as described in the next sections.

### 2.1. Source model   

The SR source used for the simulated setup has a (mean) wavelength 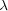 = 173 pm (17.18 keV) and a separable anisotropic Gaussian intensity profile with 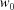 = 22.0 µm in the horizontal (H) direction and 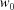 = 4.44 µm in the vertical (V) direction. The radiant intensity distribution is assumed to have a symmetric Gaussian shape, characterized by 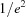 divergence angles 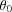 = 12.4 µrad in both H and V directions. With these choices the Gaussian Schell model is available, and the knowledge of the source dimensions and the divergence angles is sufficient for determination of the spatial coherence properties of the field (Mandel & Wolf, 1995 ▸, ch. 5.6.3). In our notation, the relation between the waist size and beam divergence is
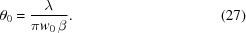
Hence, with the chosen numerical values, we have 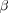 = 1 in the V direction (full spatial coherence) and 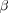 ≃ 0.2 in the H direction. We employ the elementary-field representation of the anisotropic GSM field, with a single Gaussian mode in the V direction, with 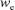 = 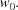 In the H direction we use equations (23) and (24) with 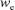 ≃ 4.4 µm and 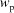 ≃ 21.5 µm. A cross section of the elementary modes used to represent this source in the source plane is shown in Fig. 1(*b*) ▸.

## 3. The simulated setup   

**Figure 2:**
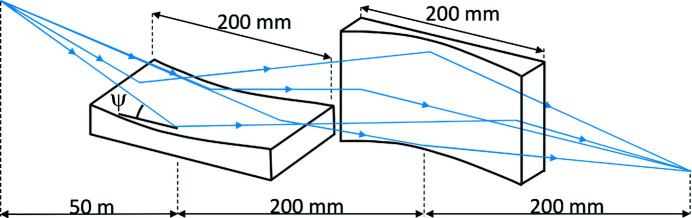
SR imaging system model with two elliptical grazing-incidence mirrors.

In the numerical simulation to be presented below we will consider an SR imaging system illustrated in Fig. 2 ▸, in which two elliptical grazing-incidence mirrors are used to form the image of a partially coherent source. The dimensions of the mirrors are 200 mm × 12 mm, their center-to-center separation is 200 mm, and they have focal lengths of 400 mm and 200 mm to enable imaging of a source located at a distance of 50 m in front of the first mirror into a distance 200 mm after the second mirror. The grazing angle (between the central tangent plane of a mirror and the local optical axis) is taken as 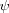 = 3 mrad.

**Figure 3:**
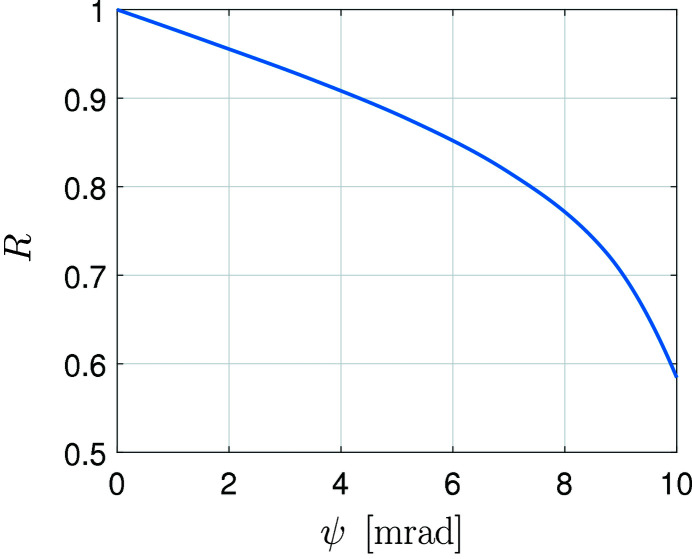
Reflectance curve used for the gold mirrors. The horizontal axis displays the grazing angle of the incoming light while the vertical axis displays the reflectance coefficient for TE-polarized light (the TM curve is practically the same).

**Figure 4:**
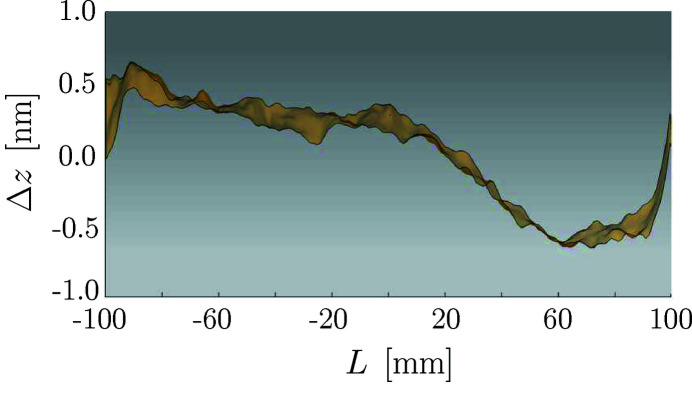
Side view of the error map (does not include mirror curvature). The mirror is 200 mm long and 12 mm wide.

The mirrors are coated with gold so that their reflectivity depends on the angle of incidence as shown in Fig. 3 ▸. The modeling of the mirrors is limited to reflective effects as simulating surface scattering would be computationally costly at this point. The mirrors surfaces are considered non-ideal and the figure errors for both mirrors are taken to be as the error-map shown in Fig. 4 ▸.

## 4. Optical-system response   

**Figure 5:**
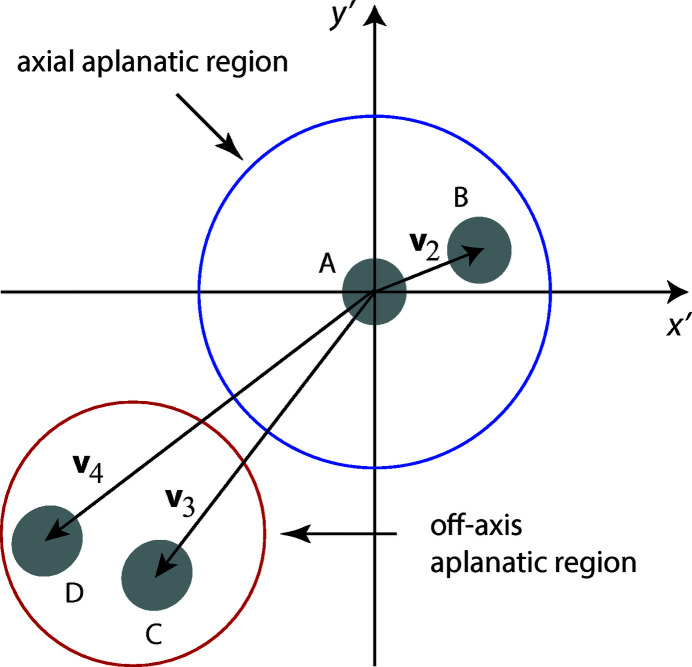
Illustration of the genuine-field representation at the input plane of an optical system.

Let us now assume that a field with a CSD of the form of equation (4) is located at the input plane of an optical system, denoting the spatial coordinates at this plane with primes. The system is, in general, considered as space-variant. For the purposes of illustration we assume for a while (as is usual in Fourier optics) that the system has an essentially aplanatic region around any point 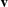 at the input 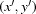 plane meaning, by definition, that the aberrations of the system remain essentially unaltered during such a region. Fig. 5 ▸ illustrates some of the genuine modal fields 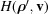, represented by the gray patches A, B, C and D. Modes A and B, centered at 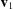 = 0 and 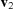, fall within the axial aplanatic region of the system. Fields C and D do not, but they are still confined within the same off-axis aplanatic region. Hence the system response is (substantially) the same for modes A and B, but different from the response for modes C and D. We will find below that the system considered has a local aplanatic region with dimensions of the same order of magnitude as the spatial extent of the genuine modal fields.

The response of a linear system is specified by its impulse response function 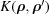, so that the CSD at the output plane is generally given by
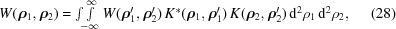
where 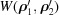 is the CSD in the input plane. If we represent the latter in the form
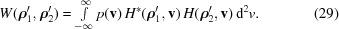
and substitute into equation (28) we obtain equation (4) with
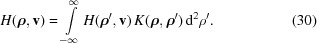
Hence also the field at the output plane is of the genuine form. By definition of an aplanatic region (Goodman, 2003 ▸) within each such region, region *K* depends only on the difference 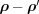.

In the numerical modeling method to be discussed in detail in Section 5 we will not make explicit use of the impulse response function but rather determine the genuine-field response 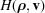 directly.

## 5. Field propagation   

Although we established that a scalar field description is appropriate for the source (Appendix *A* [App appa]), it was still conceivable that the response of the mirror system could be polarization dependent due to its three-dimensional nature and the grazing incidence on the mirrors. Since our numerical simulation software is fully vectorial in nature, we were able to establish whether this is the case.

The goal is to propagate radiation from the source to the image plane of the setup in an accurate but numerical efficient manner. This is achieved by applying a sequence of free space propagation 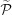 and component response operators 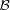 to each elementary field. These operators are applied to the (vector) field components 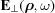 = 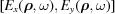 in sequence. If required, the field components 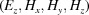 can be computed from 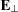 on demand (Wyrowski & Kuhn, 2011 ▸).

**Figure 6:**
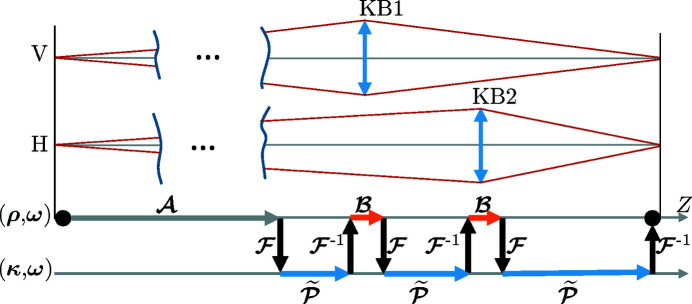
Vertical and horizontal cross sections of the setup (top) along with the field tracing diagram (bottom). The field tracing diagram shows in which region the field propagation and element response operators are applied.

Fig. 6 ▸ illustrates the field tracing diagram for the setup and shows if the operator is applied in the *x* domain [spatial, 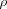 = 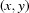] or in the *k* domain [space-frequency, 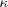 = 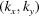]. In this diagram operator 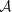 denotes the analytical solution of a Gaussian (elementary) field propagated in free space (Section 5.1). By leveraging the Fourier transform 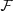 to switch between the computation domains, the free space propagation 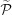 becomes a simple product operation (Section 5.2). The mirror response operator 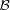 denotes the response of the grazing mirrors and is touched upon in Section 5.3.

### 5.1. Analytical source-field propagation   

Since radiation from the SR source at the plane 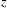 = 0 is highly paraxial, we may represent each shifted elementary field at the source in the form
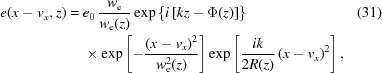
where 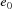 = 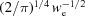,
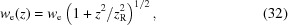


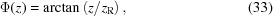


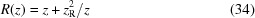
are the usual propagation parameters of a Gaussian beam (Mandel & Wolf, 1995 ▸), 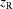 = 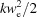 is it’s Rayleigh range, *i.e.* the distance required to increase the beam width by 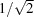, and 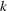 = 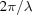. Considering the present parameter choices, the Rayleigh range in both directions is 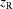 ≃ 0.35 m. Hence the switch to geometric field tracing operator 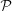 occurs in the far field of the SR source.

### 5.2. Free space propagation   

From an effort point of view it is beneficial to perform free space propagation in the *k*-domain as this simplifies the high numerical effort space domain diffraction integrals to a low effort product operation. This does require a mapping operation to switch between the domains, but this can be done with relative minimal effort by leveraging the Fourier transform in a smart manner.

Rigorous propagation in free space in the *k*-domain can be efficiently done by a simple product operation,
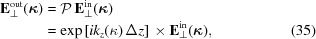
when propagating between two parallel planes. Here 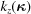 = 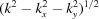. When the planes are non-parallel an additional coordinate transformation is applied (Zhang *et al.*, 2016 ▸).

To switch between the computation domains one can decompose the field in a spectrum of plane waves (SPW) by means of a Fourier transform (Goodman, 2003 ▸). If the wavefront phase is weak (*e.g.* very paraxial) this can be done accurately with relative low numerical effort. If the wavefront phase is strong this approach would require a dense sampling grid to sample the phase term accurately and could lead to unacceptable amounts of computational effort.

If this wavefront distortion is caused by a quadratic phase term, as is often the case with free space propagation, then the analytical Fourier transform can be used to minimize computation cost (Wang *et al.*, 2017 ▸). This method separates the quadratic phase term so that it does not need to be sampled while the remaining residual field can be accurately represented and mapped to the *k* domain with (much) fewer sampling points.

On the locations where the wavefront phase forms a ‘smooth’ function the mapping between the domains can be very efficiently done by use of the geometric Fourier transform (Wyrowski & Hellmann, 2017 ▸). Unlike the previous two methods this does require that the field is not located in a caustic zone.

The selection of the appropriate mapping operator is done automatically within the propagation algorithm.

### 5.3. Mirror response operator   

The mirror response operators involve applying the Kirchhoff’s boundary conditions and solving the appropriate electromagnetic boundary conditions at the mirror interface. The mirror operator 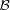 can be computed with minimal cost in the *x* domain and thus the appropriate inverse Fourier transform is applied after propagation to represent the field in the *x* domain as well.

**Figure 7:**
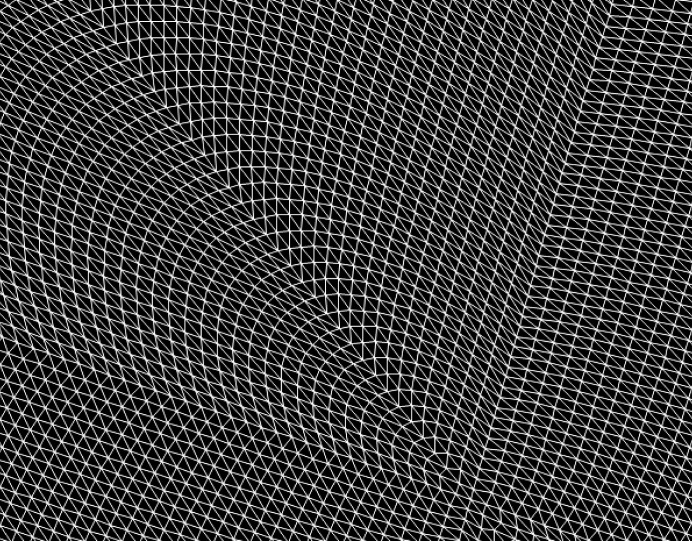
Local plane wave approximation. Each three adjacent points denote the corners of a local plane wave. From this representation the full vectorial field can be reconstructed as long as adjacent (local) plane waves do not overlap (*i.e.* no diffraction effects).

In general the interaction of a field with a surface can be obtained by use of rigorous Maxwell solvers. If the surface is smooth and its structures not too small the interaction of the field with the surface can be much more efficiently modeled by means of the Local Plane Interface Approximation (LPIA). Under this method the surface is locally treated as a plane interface. If the field is also represented as a patchwork of local plane waves, which can be done as the phase is assumed to be smooth, then the element’s response is described by a plane-wave plane interface interaction (Pfeil *et al.*, 2000 ▸). A visualization of the local plane wave approximation is shown in Fig. 7 ▸.

## 6. Numerical results   

In this section we provide simulation results for KB mirrors performed using *VirtualLab* software (LightTrans GmbH, 2015 ▸) with and without figure errors. We begin by assuming that the source is fully coherent also in the H direction, with a far-field divergence angle of 12.4 µrad. Then the effect of partial coherence is discussed by means of the genuine superposition of all elementary-field contributions in the image plane.

The discussion in Appendix *A* [App appa] shows that the scalar model is adequate for SR field description in free space, but it does not prove this to be the case in propagation through the KB mirror system. However, our numerical model, which traces all components of the electromagnetic field through the system, does so. By assuming a horizontally linearly polarized source, we found that the *y* and *z* components (combined) of the electric field in the image plane contain less than 0.01% of the total energy. Hence, when plotting the results, we consider only the *x* component of the electric field and conclude that the intensity profiles thus obtained have the same shape (within plotting accuracy) regardless of the state or degree of polarization of the source.

### 6.1. Coherent source   

**Figure 8:**
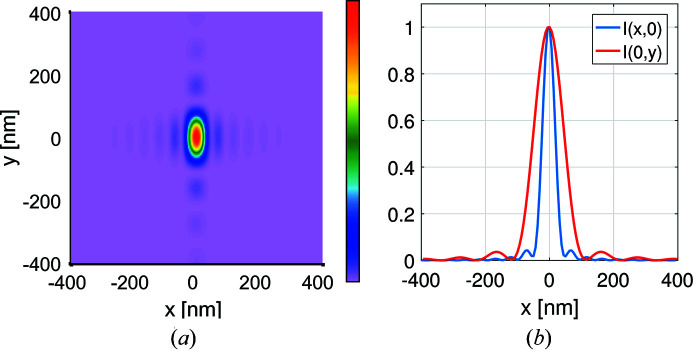
The focal spot (*a*) and its cross section (*b*) when using a coherent Gaussian source with an opening angle of 12.4 µrad for ideal mirrors and for mirrors with figure errors (*c*, *d*). The intensity plots were normalized so as to contain the same energy as the coherent source for the ideal mirrors case. By comparison the aberrated mirrors results in a maximum amplitude of 0.992 if for the ideal mirrors amplitude is normalized to reach unity.

After 50 m of propagation the coherent Gaussian field has a (FWHM) cross section of about 732 µm × 735 µm (H, V). Compared with the effective cross section 716 µm × 624 µm of the mirrors (see Appendix *B* [App appb]), this means that the mirrors clip the field in both directions, but more strongly along the vertical direction. From this one would expect to see something between Gaussian and sinc profiles in the horizontal direction, and something closer to a sinc profile along the vertical direction as is indeed the case as shown in Fig. 8 ▸. The Rayleigh spot size is 50.0 ± 0.5 nm × 116 ± 0.5 nm (H, V) which is slightly larger than the 48.3 nm × 111 nm expected spot size. The difference from ideal is attributed to the non-uniform illumination in the effective window caused by the Gaussian intensity profile and grazing angle dependence of the reflectance coefficient. All figures in the results section have been normalized with the same normalization constant as used in the coherent-ideal mirrors case shown in Figs. 8(*a*) and 8(*b*) ▸.

### 6.2. Partially coherent source   

The intensity of the genuine-field superposition due to all elementary-field contributions 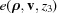 in the focal plane of the mirror system is given by
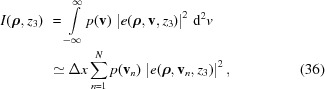
with the weight function defined by equation (24). In the discrete representation 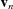 = 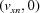, and the sampling points 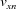 = 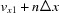 are chosen equidistantly to cover the extent of the function 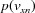. To keep the error <0.5% we used 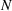 = 17 elementary modes with 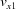 = 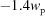 and 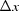 = 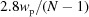 so as to obtain good convergence of the results for both ideal mirrors and mirrors with figure errors. Doubling the number of modes showed no significant change in the results.

**Figure 9:**
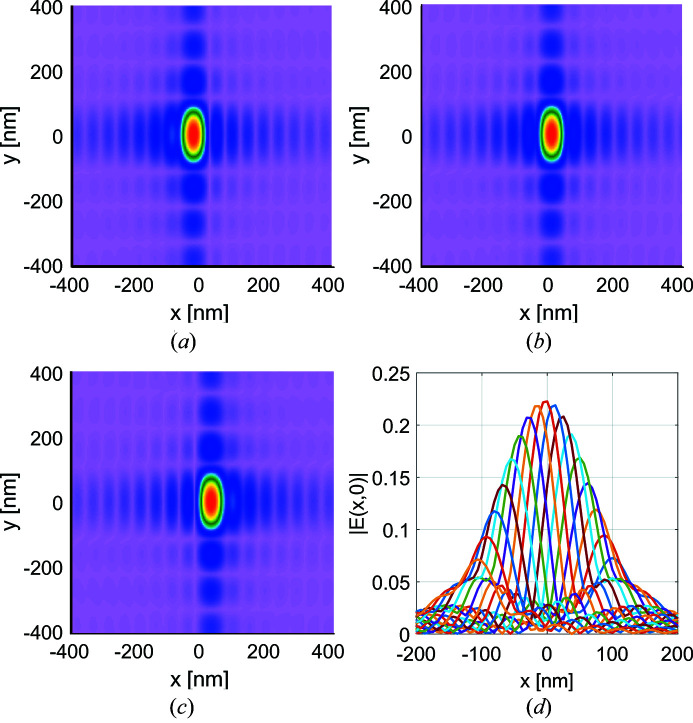
Electric field amplitudes 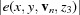 due to elementary fields 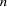 = 7 (*a*), 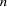 = 9 (*b*) and 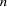 = 11 (*c*) for a system with ideal mirrors at the focal plane of the mirrors. Panel (*d*) shows the *x* cross sections of all image-plane modes produced by elementary fields 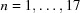.

Fig. 9 ▸ shows the absolute values of the *x*-components of the image-plane genuine field modes 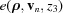 for 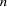 = 7, 9, 11, in the case of ideal mirrors. We plot the absolute values of fields (instead of intensity distributions) to better illustrate the sidelobe structure. The odd-numbered modes were chosen to better see the otherwise small difference between adjacent modes in the electric field amplitude plots. In all figures the total field was normalized such that they contain the same energy as the fully coherent source shown in Fig. 8 ▸. Inspection of the fields with different values of *n* shows that the functional form of the elementary modes slightly differentiate from one another, indicating that the size of the local aplanatic region of the system (*cf.* Fig. 5 ▸) is comparable with the spatial extent of the image-plane spot, even though the system is highly paraxial. The small variations in the shape of adjacent modes indicate that sufficient elementary modes were used to sample the space-variant response of the system.

**Figure 10:**
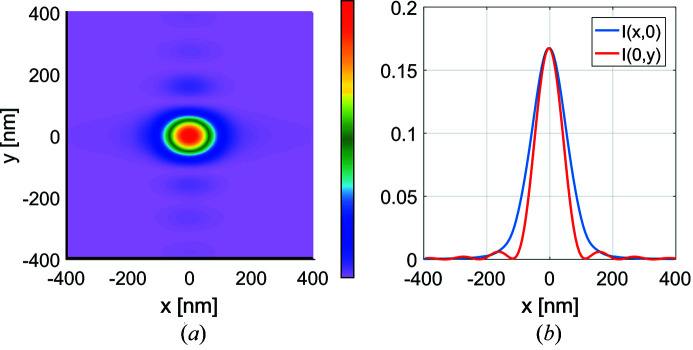
Intensity distribution of the focal spot with a partially coherent source in the case of ideal mirrors.

Fig. 10 ▸ illustrates the total intensity distribution given by equation (36) in the case of ideal mirrors. As expected, the intensity cross section in the vertical direction remains nearly the same as in the fully coherent case. However, as the source is now partially coherent in the horizontal direction, the incoherent superposition of different genuine image-plane modes in equation (36) virtually smooths out the effect of apodization in this direction: the sidelobes of the *x* cross section of the total intensity profile almost disappear.

**Figure 11:**
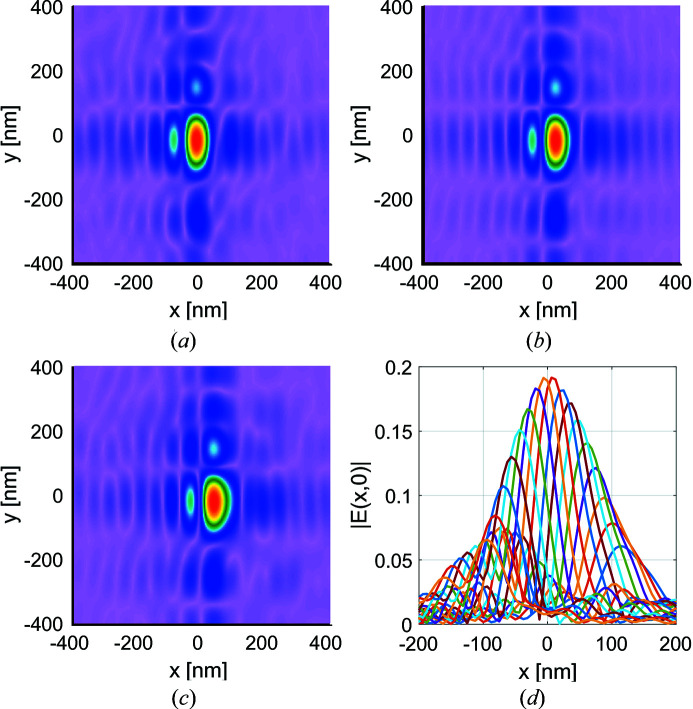
Same as Fig. 9 ▸, but for a system with five times the figure errors. The intensity cross sections of all elementary modes is shown in the final plot.

**Figure 12:**
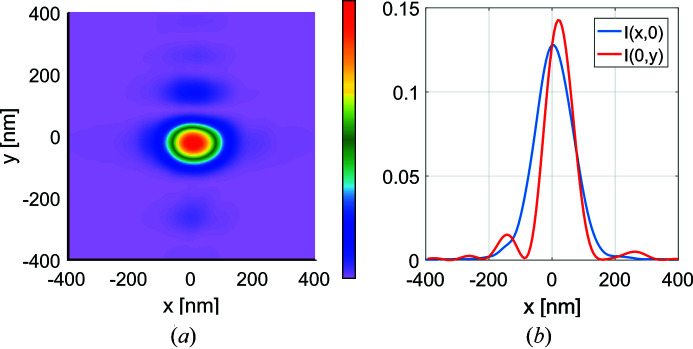
Intensity distribution of the focal spot with a partially coherent source in the case of a system with five times the figure errors.

Due to the small abberations the results for mirrors with figure errors deviate only slightly from the ideal mirrors results. As such these figures are omitted and five times the error map are shown instead in Figs. 11 ▸ and 12 ▸ to demonstrate how significant errors would affect the focal spot. Fig. 11 ▸ shows the amplitude plots of some modes out of the total 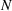 = 17. On comparison with Fig. 9 ▸, we see that mode-to-mode variations are greater for non-ideal mirrors. In Fig. 12 ▸ the intensity at the focus is shown, where due to the abberations on the mirror the focal spot has moved slightly off-center.

**Figure 13:**
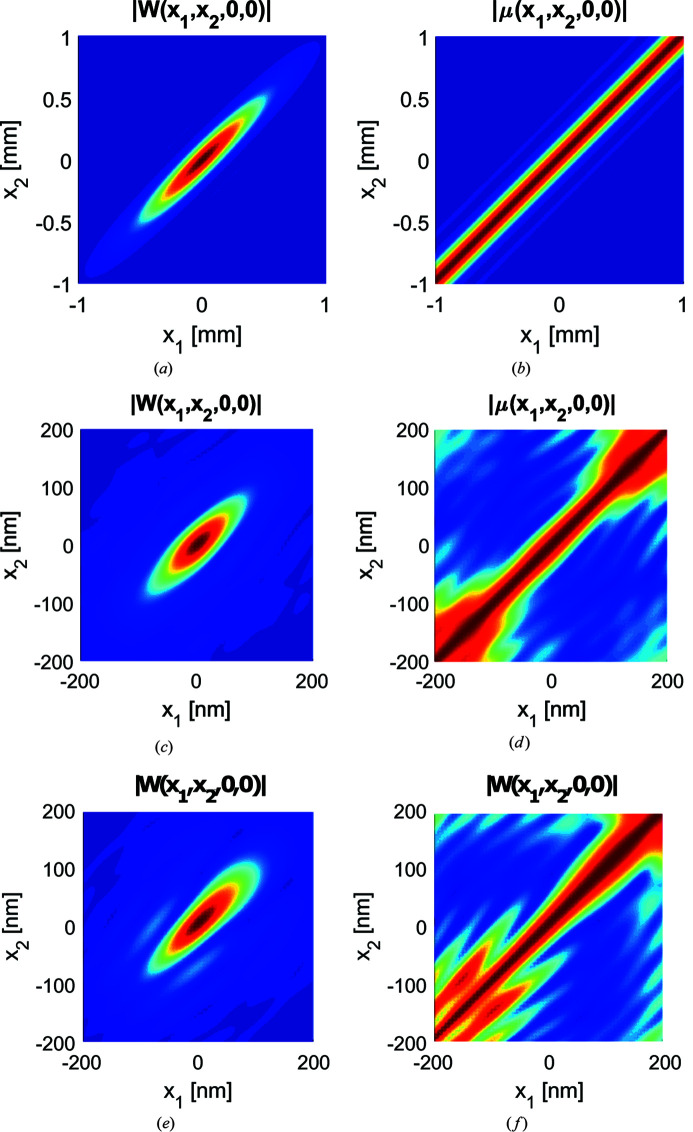
Distribution of the CSD (left) and the complex degree of spatial coherence (right) along the *x* cross section of the source right before the mirrors (top row), image-plane field for the ideal mirrors (middle row) and mirrors with figure errors (bottom row).

The distributions of absolute values of the *x*-directional cross-spectral density function 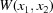 and the associated complex degree of spatial coherence
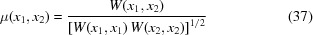
are shown in Fig. 13 ▸ for systems with ideal and non-ideal mirrors [it should be noted that the computation of 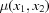 is poorly conditioned in regions where the intensity 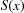 = 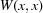 of the field is low]. The effective width of 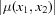 in the antidiagonal direction 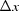 = 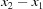 represents the local coherence width of the field at average position 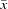 = 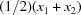 and the field is of the Schell-model form if the coherence width is independent on 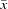. Clearly, this is not the case in the image plane, though the field at the source plane is of the Schell-model form. The coherence width increases with 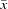 for systems with both ideal and non-ideal mirrors. However, it remains essentially constant in the region of high intensity (along the diagonal line) especially for the system with ideal mirrors.

## 7. Conclusion and outlook   

A new method for beamline simulation based on wavefront propagation is described. These very powerful techniques can be used to simulate X-ray optics under fully or partially coherent illumination. This method is accurate, fast and can be used for predicting the X-ray focusing performance of X-ray focusing mirrors.

The next step is to create and include a quadratic phase extraction operator that facilitates the switch between field propagation operators after hitting an aperture. This allows simulating more complex X-ray beamlines like various X-ray microscope or soft X-ray beamlines and compare said simulations with real measurements. Another refinement would be to replace the Gaussian elementary mode with the true single-electron radiation mode (Khubbutdinov *et al.*, 2019 ▸), which would readily lead to an accurate partially coherent source model. We expect further applications of these interesting simulation tools.

## Figures and Tables

**Figure 14:**
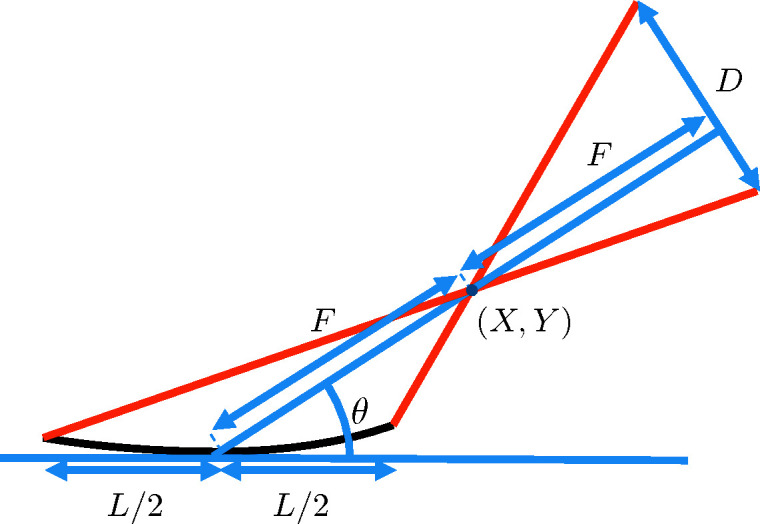
Cross-sectional sketch of a grazing-incidence mirror of size *L*, with the focus located at 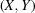.

**Figure 15:**
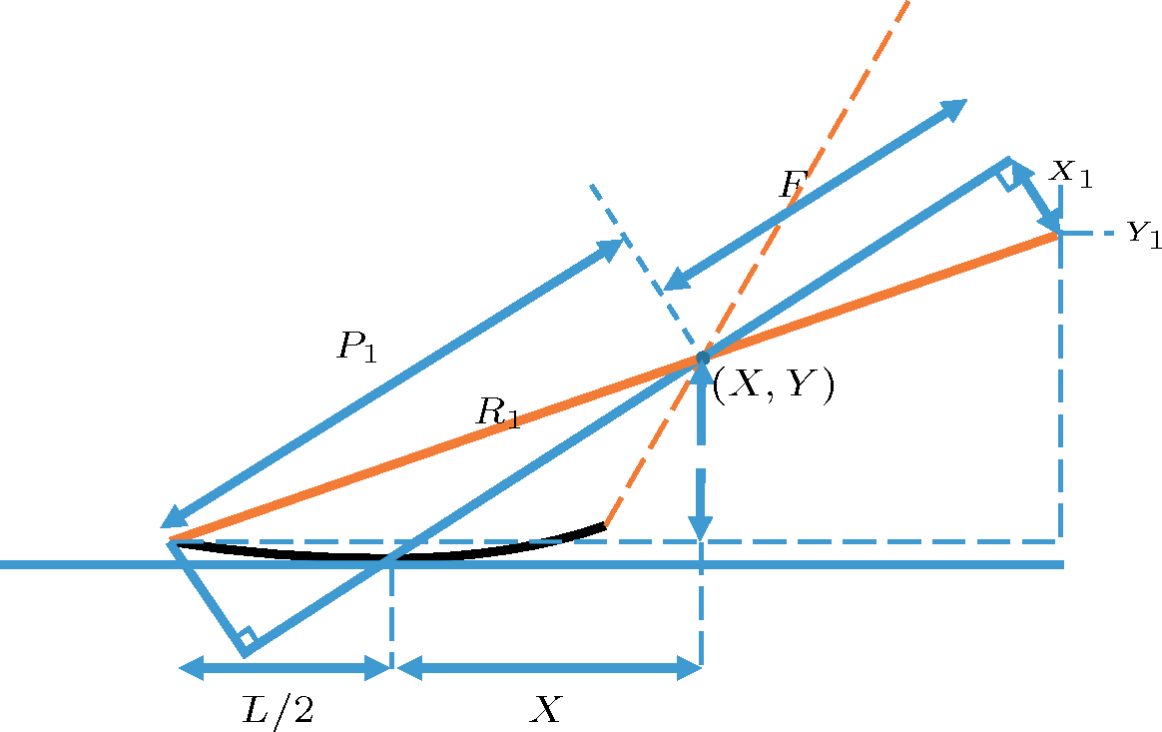
Same as Fig. 14 ▸, but with some visual aid for computing 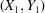.

**Figure 16:**
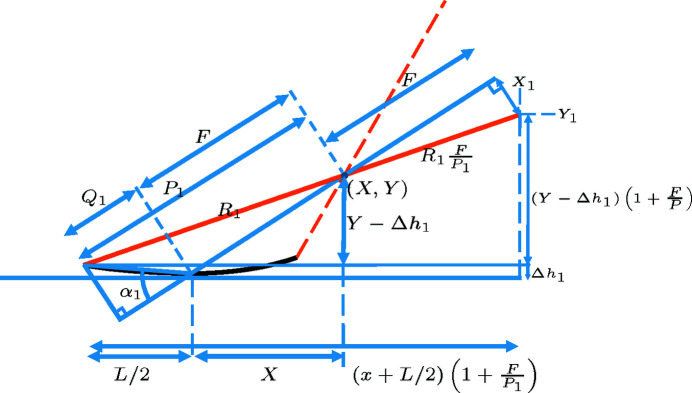
Same as Fig. 15 ▸, but with more visual aid for computing 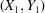.

**Figure 17:**
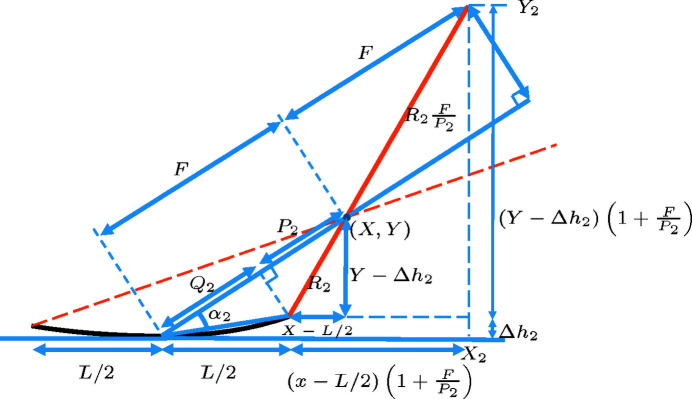
Same as Fig. 15 ▸, but with some visual aid for computing 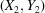.

## Appendix A Justification of the field description

Let us consider a non-stationary (pulsed) random electromagnetic field with space–time domain electric field realizations . The second-order coherence properties of the field in the space–time domain are then fully described by the two-time mutual coherence matrix  = , where  and  are positions vectors in a transverse plane  = constant, the angular brackets  indicate an ensemble average over all realizations,  denotes the complex conjugate, and T denotes the transpose. Correspondingly, second-order coherence in the space–frequency domain is characterized by the cross-spectral density (CSD) matrix  = , where

are the space–frequency domain realizations of the electric field. In general the correlation matrices are  matrices, but for paraxial fields (such as X-ray beams) they reduce to  coherence-polarization matrices (Gori, 1998 ▸; Gori *et al.*, 1998*b* ▸; Wolf, 2003 ▸) because the longitudinal field components are insignificant.

If the polarization statistics of the field are spatially uniform across the beam and we use the envelope representation of the temporal field, we may write  = , where  is a random (fast-varying) polarization vector,  can be taken as the mean optical frequency of the spectrum, and  is the scalar field envelope. If the beam is quasimonochromatic, as pulsed X-ray beams typically are, the envelope varies slowly in time compared with field oscillations at the mean frequency . Under these conditions we can consider  and  as statistically independent quantities and write  as a product of a  matrix  =  and a scalar MCF

In the case of SR it is reasonable to assume that the polarization properties of the field do not change appreciably over the temporal duration of the pulse, allowing us to approximate  =  = , where  is a constant polarization matrix of the beam. Propagation in polarization-insensitive optical systems does not modify , and therefore it is sufficient to consider only the propagation of the scalar MCF defined in equation (39), or the corresponding scalar CSD

where  is defined in analogy with equation (38).

Let us next introduce average and frequency temporal coordinates  =  and  = , and assume that the scalar MCF can be factored in the form

Here the function  =  represents the spatial coherence between light fluctuations at points  and , and in general it can depend on time. A non-stationary field is called quasi-stationary if, for any pair of spatial points and at any , the absolute value of the quantity

is a narrow function of  compared with the temporal intensity distribution  =  at point . For fields of the form of equation (42) we may approximate  = , where

is the time-dependent (complex) degree of spatial coherence of the field. On the other hand, introducing average and difference frequency coordinates  =  and  = , we have from equation (40)

where

and

Clearly, because of the Fourier transform relationships,  is a wide function compared with . The effective width of the absolute value of the latter function is a measure of the spectral coherence width, which is small compared with the bandwidth of the power spectrum,  = , at position . Hence we may write

where

and

is the spatial part of the CSD evaluated at the center frequency . This is the quantity we employ in this paper to study the spatial coherence of SR; for brevity we drop the subscript S in the main text.

It appears intuitively inconceivable that the spatial coherence properties of the field could change appreciably over a time scale larger than the coherence time, which for quasi-stationary fields is so short that no substantial changes are likely to occur within this time scale either. Hence we may write  =   . Correspondingly, it is not likely that spatial coherence is the space–frequency domain will change appreciably outside the frequencies within spectral coherence bandwidth of the field, which approaches zero in the fully stationary limit.

In view of the considerations presented above, the scalar representation of spatial coherence with the aid of a CSD evaluated at  =  requires a large number of assumptions, some of which are difficult to quantify. It is, for example, not difficult to construct (at least mathematically) pulsed beams with femtosecond-scale temporal variations of the coherence-polarization matrix (we do not dwell deeper into such constructions here). However, it appears that there are no physical mechanisms in SR generation that would lead to such ultrashort modulation. The assumption that SR is quasi-stationary is well justified. For example, coherence times of the order of 3–6 fs were measured for X-ray free-electron laser pulses with duration of ∼30 fs (Roling *et al.*, 2011 ▸). The assumption that the MCF can be written in a factored form as indicated in equation (42) is an approximation that cannot be made in general, though it appears safe to do so for SR. The spatial scale of the CSD of non-stationary fields is normally a function of the two frequencies involved, and a function of frequency also for stationary fields. Including such frequency dependence would, via Fourier inversion of equation (40), immediately lead to space–time coupling effects and thereby a violation of the factorization condition (42). Recent model studies (Koivurova *et al.*, 2018 ▸) indicate, however, that these effects are significant only in the few-cycle regime and become less apparent when the degree of spatial coherence of the pulsed beam is reduced.

## Appendix B Resolution limit of KB mirrors

The standard Rayleigh limit of an on-axis lens with a rectangular aperture of size  and focal length *F* is  = . The KB mirror system can be modeled as having an effective rectangular aperture of size *D* and focal length *F* in both horizontal and vertical directions (though these are different), but the calculation of the effective *D* requires some consideration of the grazing-incidence geometry. It turns out that the Rayleigh resolution of the KB mirror system is somewhat higher than the result one would expect by application of the Rayleigh limit to the apparent size of the mirrors.

To compute the Rayleigh limit for grazing-incidence geometry we consider the geometry of Fig. 14 ▸ which shows a KB mirror with grazing angle θ (greatly exaggerated for clarity) and back focal distance *F*. We place the origin at the center of the mirror and assume that the focus is located at position  with  =  and  = . The red lines are the two marginal rays reflected from the front and rear edges of the mirror and pass through the focus. The effective window size of the mirror is the separation *D* of these rays at a distance *F* behind the focus, measured perpendicularly to the local optical axis.

To compute *D* we need to determine the coordinates  and  of the points where the marginal rays cross the line perpendicular to the optical axis. Fig. 15 ▸ contains some additional notation to make the reasoning easier to follow. Two similar sets of triangles can be identified, one set with solid and another set with dashed lines. They both share the same slope indicated by the orange line. The slope of both sets of triangles (orange line) consists of two sections with lengths  and . If the length of  is known, the solid similar triangles can be used to express  as

From this relationship the location  can be expressed by using the known sizes of the smaller and larger dashed triangle,

where  is the ratio by which the larger triangle is larger then the smaller triangle with height  inside it.

To compute , Fig. 15 ▸ is re-drawn as shown in Fig. 16 ▸. From here we see that

where

Now all information is know to compute .

Among similar lines the location of the upper edge  can be computed using the notation indicated in Fig. 17 ▸. From here we find that

with

so that the effective window size is given by

To compute the effective window size for the system considered here we insert  = 3 mrad,  = 0.173 nm,  = 200 mm, and  = 200 mm (horizontal focus) or  = 400 mm (vertical focus) into the expressions derived above. The heights of the edges are  = 30 µm,  = 52 µm (horizontal mirror) and  = 17 µm,  = 22 µm (vertical mirror). These values give an effective window size of  = 716 µm in the horizontal direction and  = 624 µm in the vertical direction. These values give a Rayleigh resolution limit of 48.3 nm × 111 nm (H, V), which is smaller than one would obtain by a simple approximation (57.7 nm × 115 nm).
